# The impact of value-based leadership on employee innovative behavior: the mediating role of organizational identification and moderating role of person-organization matching

**DOI:** 10.3389/fpsyg.2026.1807510

**Published:** 2026-06-02

**Authors:** Rongcheng Liang

**Affiliations:** 1 Emergency Management Training Department, Party School of Shandong Provincial Committee of the Communist Party of China (Shandong Academy of Governance), Jinan, China; 2 School of International Relations and Public Affairs, Fudan University, Shanghai, China

**Keywords:** employee innovative behavior, organizational identification, person-organization matching, social exchange theory, value-based leadership

## Abstract

Drawing upon social exchange theory and self-determination theory, this study investigates the mechanisms through which value-based leadership is associated with employee innovative behavior. Specifically, this study examined organizational identification as a mediator and person-organization matching as a moderator in this relationship. Using a sample of 412 employees from various organizations in China, this study conducted hierarchical regression analyses to test our hypotheses. The results reveal that value-based leadership significantly and positively predicts employee innovative behavior, and this relationship is partially mediated by organizational identification. Furthermore, person-organization matching moderates the relationship between value-based leadership and organizational identification, such that the positive effect is stronger when person-organization matching is high. Additionally, person-organization matching moderates the indirect effect of value-based leadership on innovative behavior via organizational identification. These findings contribute to the leadership and innovation literature by elucidating the psychological mechanisms and boundary conditions of value-based leadership’s influence on employee innovation. Practical implications for organizations seeking to foster innovative behavior through value-based leadership are discussed.

## Introduction

In today’s rapidly changing business environment, characterized by globalization, technological disruption, and intensifying competition, innovation has become a critical determinant of organizational success and competitive advantage (Tran Pham, 2026a, 2026b). Organizations across industries increasingly rely on their employees’ innovative behavior to adapt to market changes, develop new products and services, improve operational processes, and maintain sustainable growth in an ever-evolving landscape. Employee innovative behavior refers to the intentional creation, introduction, and application of new ideas within a work role, work group, or organization, encompassing idea generation, idea promotion, and idea realization (Tamasevicius et al., 2026). Therefore, understanding the factors that promote or inhibit employee innovative behavior has thus become a central concern for both researchers and practitioners seeking to foster organizational innovation.

Among these factors, leadership has consistently emerged as one of the most influential determinants of employee innovative behavior (Nguon, 2022; Tran Pham, 2026b; Almutairi et al., 2025; Zhang et al., 2015), shaping the psychological conditions and contextual factors that either facilitate or constrain employees’ willingness and ability to engage in innovative activities (Nazir and Khadim, 2026; Bindel Sibassaha et al., 2025; Irayani et al., 2025; Basahel, 2021). Among various leadership styles, value-based leadership has garnered considerable attention in recent years due to its emphasis on aligning organizational values with employee personal values (House et al., 2002). Value-based leaders articulate a clear vision, communicate core values consistently, and inspire followers to transcend self-interest for the collective good. This leadership approach is rooted in the broader transformational leadership paradigm but places particular emphasis on the role of values in motivating and inspiring followers (Kraemer, 2025; Grant, 2012). Despite the growing interest in value-based leadership, our understanding of how it influences employee innovative behavior remains limited and fragmented. While some studies have demonstrated positive relationships between value-based leadership and various employee outcomes (Hendrickson and Blackmon, 2025), the specific mechanisms through which value-based leadership translates into innovative behavior are not well understood. Furthermore, the boundary conditions that strengthen or weaken these relationships require further investigation to provide practical guidance for organizational leaders.

To address these gaps, this study draws upon social exchange theory and self-determination theory to develop a comprehensive moderated mediation model that elucidates the mechanisms and boundary conditions of value-based leadership’s association on employee innovative behavior. Notably, while social exchange theory emphasizes extrinsic reciprocity mechanisms, self-determination theory focuses on intrinsic need satisfaction; these perspectives offer complementary yet distinct explanations for how leadership fosters innovation, a theoretical tension that prior research has largely overlooked. Social exchange theory provides a framework for understanding how the reciprocal exchange relationship between leaders and followers can motivate employees to engage in innovative behaviors as a form of reciprocation for the leader’s value-based support and inspiration. Self-determination theory complements this perspective by explaining how value-based leadership can satisfy employees’ basic psychological needs, thereby fostering intrinsic motivation for innovation. Social exchange theory provides a framework for understanding how the reciprocal exchange relationship between leaders and followers can motivate employees to engage in innovative behaviors as a form of reciprocation for the leader’s value-based support and inspiration. Self-determination theory complements this perspective by explaining how value-based leadership can satisfy employees’ basic psychological needs, thereby fostering intrinsic motivation for innovation. This study proposes organizational identification as a key mediator that explains how value-based leadership fosters employee innovative behaviors. Additionally, this study examines person-organization matching as a moderator of the relationship between value-based leadership and organizational identification. Person-organization matching reflects the compatibility between individual values and organizational values (Kristof-Brown et al., 2005).

This study makes several important contributions to the leadership and innovation literature. First, by examining organizational identification as a mediator, it illuminates the psychological mechanisms through which value-based leadership influences employee innovative behavior, extending previous research that has primarily focused on direct relationships without exploring the underlying processes. Second, by investigating person-organization matching as a moderator, this study identifies important boundary conditions that shape the effectiveness of value-based leadership, providing a more nuanced understanding of when and for whom value-based leadership is most effective. Third, by integrating social exchange theory and self-determination theory, this study offers a comprehensive theoretical framework that can guide future research on leadership and innovation. Fourth, our findings provide practical guidance for organizations seeking to enhance employee innovative behavior through value-based leadership practices and value-aligned human resource management strategies.

## Theoretical background and hypotheses

### Value-based leadership and employee innovative behavior

Value-based leadership is a leadership approach that emphasizes the articulation and embodiment of core values to inspire and motivate followers (House et al., 2002). The GLOBE study, a comprehensive cross-cultural investigation of leadership across 62 societies, identified value-based leadership as a universally endorsed leadership attribute that contributes to outstanding leadership across cultural contexts (House et al., 2002). Innovative behavior is critical for organizational adaptation and success in dynamic environments, as it enables organizations to respond effectively to changing market conditions, technological developments, and competitive pressures (Tran Pham, 2026a). This study found that value based leadership has a positive impact on employees’ innovative behavior.

Social exchange theory provides a theoretical foundation for understanding how value-based leadership influences employee innovative behavior. According to this theory, social relationships develop through a process of reciprocal exchanges, where individuals feel obligated to reciprocate beneficial treatment they receive from others. Unlike economic exchanges, which involve explicit contracts and tangible rewards, social exchanges involve implicit obligations, trust, and mutual commitment. When leaders demonstrate value-based behaviors, employees perceive this as a form of social support and are motivated to reciprocate through positive work behaviors, including innovative behavior. The social exchange relationship between leaders and followers is particularly relevant for understanding innovative behavior because innovation often involves going beyond formal job requirements and taking risks that may not be explicitly rewarded. When employees perceive that their leaders genuinely care about their well-being and act in accordance with shared values, they develop a sense of obligation to reciprocate through discretionary behaviors that benefit the organization, such as generating and implementing innovative ideas. This reciprocation mechanism is central to understanding how value-based leadership can foster employee innovative behavior.

Furthermore, self-determination theory suggests that value-based leadership can satisfy employees’ basic psychological needs for autonomy, competence, and relatedness, thereby fostering intrinsic motivation for innovative behavior. Autonomy refers to the need to experience choice and volition in one’s actions. By articulating a clear vision and empowering employees to contribute to that vision, value-based leaders foster a sense of autonomy that encourages creative exploration and experimentation. Competence refers to the need to feel effective in one’s activities. By recognizing and appreciating employees’ contributions, value-based leaders enhance feelings of competence that support innovative efforts. Relatedness refers to the need to feel connected to others. By creating a values-based community, value-based leaders satisfy the need for relatedness that encourages collaborative innovation.

Empirical evidence supports the positive relationship between value-based leadership and employee outcomes. For example, studies have shown that value-based leadership is associated with increased employee engagement, organizational commitment, job satisfaction, and organizational citizenship behavior (Kraemer, 2025). Recent research has also begun to explore the relationship between value-based leadership and employee innovative behavior, finding positive associations that suggest value-based leadership can create the psychological conditions necessary for innovation (Tirmizi et al., 2023). Building on this literature and drawing upon social exchange theory and self-determination theory, it proposes:

*H1*: Value-based leadership is positively related to employee innovative behavior.

### Organizational identification as a mediator

Organizational identification has been shown to have important implications for employee attitudes and behaviors. Employees who strongly identify with their organization are more likely to engage in organizational citizenship behavior, exhibit lower turnover intentions, and demonstrate higher levels of job satisfaction and organizational commitment (Tadena, 2026). From a social identity perspective, organizational identification provides a basis for self-definition that extends beyond individual attributes to include group membership, creating a sense of shared fate and collective purpose.

This study proposes that value-based leadership enhances organizational identification through several mechanisms. First, value-based leaders articulate and embody organizational values, making them salient and meaningful to employees. When employees perceive that their leaders genuinely believe in and act upon organizational values, they are more likely to internalize these values and develop a sense of identification with the organization. Second, value-based leaders create a shared sense of purpose that connects individual work to broader organizational goals, fostering a collective identity that strengthens organizational identification. Third, value-based leaders demonstrate integrity and authenticity, which builds trust and facilitates the identification process.

Organizational identification, in turn, promotes employee innovative behavior through multiple pathways. Employees who strongly identify with their organization are more likely to engage in behaviors that benefit the organization, including innovative behavior, because they view organizational success as personally relevant and experience organizational outcomes as self-relevant. This psychological connection creates a sense of psychological ownership over organizational outcomes, motivating employees to go beyond their formal job requirements to contribute to organizational effectiveness through innovative efforts. Additionally, organizational identification enhances employees’ willingness to take risks and experiment with new ideas, as they feel secure in their relationship with the organization and trust that their innovative efforts will be valued and supported. Employees who identify strongly with their organization are also more likely to engage in proactive behaviors, such as scanning the environment for improvement opportunities and championing new ideas, because they are psychologically invested in the organization’s success. Furthermore, organizational identification facilitates collaborative innovation by fostering a sense of shared purpose and collective identity that encourages knowledge sharing and joint problem-solving.

Empirical research supports the mediating role of organizational identification in leadership-outcome relationships. For instance, organizational identification mediates the relationship between transformational leadership and employee outcomes such as organizational citizenship behavior and job performance (Walumbwa et al., 2009). Similarly, research has shown that organizational identification mediates the relationship between ethical leadership and employee outcomes, suggesting that identification is a key mechanism through which leadership influences employee behavior. Based on this theoretical reasoning and empirical evidence, it proposes:

*H2*: Value-based leadership is positively related to organizational identification.

*H3*: Organizational identification mediates the relationship between value-based leadership and employee innovative behavior.

### Person-organization matching as a moderator

High person-organization matching occurs when employees perceive that their personal values align with organizational values, creating a sense of congruence that enhances work attitudes and behaviors. Person-organization matching has been shown to have important implications for employee attitudes, behaviors, and organizational outcomes. Meta-analytic evidence indicates that person-organization fit is positively associated with job satisfaction, organizational commitment, and job performance, and negatively associated with turnover intentions (Kristof-Brown et al., 2005).

This study proposes that person-organization matching moderates the relationship between value-based leadership and organizational identification. Drawing upon person-environment fit theory and leadership contingency perspectives, it argue that value congruence serves as a contextual amplifier that enhances leadership effectiveness. When employees perceive strong alignment between personal and organizational values, they become more receptive to value-based leadership messages, thereby deepening their identification with the organization. When person-organization matching is high, employees already perceive alignment between their personal values and organizational values. In this context, value-based leadership reinforces and validates this alignment, strengthening the positive effect on organizational identification. The leader’s emphasis on organizational values resonates with employees who share those values, creating a powerful sense of connection and identification. The consistency between the leader’s value-based messages and the employee’s personal values facilitates the internalization process and strengthens the identification with the organization. Conversely, when person-organization matching is low, employees perceive a disconnect between their personal values and organizational values. In this situation, value-based leadership may be less effective in fostering organizational identification, as employees may view the leader’s value-based messages as inconsistent with their own beliefs and priorities. The misalignment between personal and organizational values creates cognitive dissonance that weakens the relationship between value-based leadership and organizational identification. Employees who experience value incongruence may be more resistant to leader influence and less likely to internalize organizational values, limiting the effectiveness of value-based leadership in fostering identification.

Furthermore, person-organization matching may moderate the indirect effect of value-based leadership on employee innovative behavior through organizational identification. When person-organization matching is high, the mediating pathway through organizational identification should be stronger, as value-based leadership more effectively fosters identification, which in turn promotes innovative behavior. When person-organization matching is low, this mediating pathway should be weaker. Based on this reasoning, it proposes:

*H4*: Person-organization matching moderates the relationship between value-based leadership and organizational identification, such that the positive relationship is stronger when person-organization matching is high.

*H5*: Person-organization matching moderates the indirect effect of value-based leadership on employee innovative behavior through organizational identification, such that the indirect effect is stronger when person-organization matching is high.

### Conceptual framework

**Figure 1:**
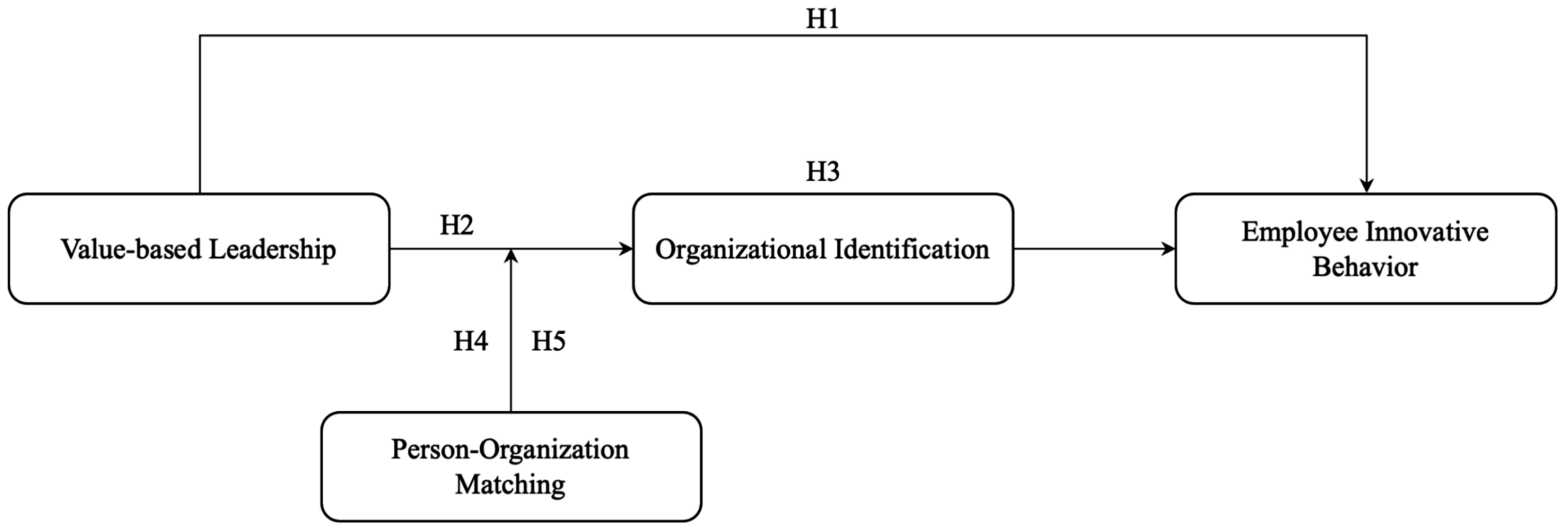
Research framework.

Figure 1 presents the conceptual framework of this study, illustrating the hypothesized relationships among value-based leadership, organizational identification, person-organization matching, and employee innovative behavior. The model proposes that value-based leadership positively influences employee innovative behavior both directly and indirectly through organizational identification. Additionally, person-organization matching moderates the relationship between value-based leadership and organizational identification, as well as the indirect effect on innovative behavior.

## Materials and methods

### Sample and procedure

This study collected data from employees working in various organizations across different industries in China, including technology, manufacturing, finance, and services. Given resource constraints, it employed a convenience sampling approach, recruiting participants through professional networks and online platforms across multiple cities in China. The time for collecting data and conducting further research was from June 2023 to June 2024, and detailed modifications were made to the research afterwards. All study variables—including value-based leadership, organizational identification, person-organization matching, and employee innovative behavior—were conceptualized and measured at the individual level of analysis. To ensure data quality and reduce common method bias, it employed a two-wave data collection design with a four-week interval between the two waves (Podsakoff et al., 2003).

In Time 1, it distributed questionnaires to 600 employees, measuring value-based leadership, person-organization matching, and demographic variables. Four weeks later, in Time 2, it sent follow-up questionnaires to the same employees, measuring organizational identification and employee innovative behavior. This temporal separation helps establish causal precedence and reduces the influence of transient mood states on responses.

Of the 600 employees who received the Time 1 questionnaire, 512 completed and returned it (response rate = 85.3%). Of these, 412 also completed the Time 2 questionnaire, resulting in a final sample of 412 matched responses (retention rate = 80.5%). The final sample consisted of 228 males (55.3%) and 184 females (44.7%). The average age was 34.2 years (SD = 7.8), and average organizational tenure was 6.4 years (SD = 4.2). In terms of education, 37.9% had a bachelor’s degree, 48.0% had a master’s degree, and 14.1% had a doctoral degree or other advanced qualifications. Table 1 presents the detailed sample characteristics.

**Table 1:** Sample characteristics (*N* = 412).

Characteristic	Category	*N*	Percentage (%)
Gender	Male	228	55.3
	Female	184	44.7
Age	Under 30	98	23.8
	31–40	186	45.1
	41–50	98	23.8
	Over 50	30	7.3
Education	Bachelor	156	37.9
	Master	198	48.0
	Doctorate	58	14.1
Tenure	Less than 3 years	86	20.9
	3–5 years	124	30.1
	5–10 years	142	34.5
	More than 10 years	60	14.5
Position level	Junior staff	142	34.5
	Middle management	186	45.1
	Senior management	84	20.4

### Measures

#### Value-based leadership

It measured value-based leadership using a 15-item scale adapted from House et al. (2002). Sample items include “My leader articulates a clear vision for the organization” and “My leader acts in ways that reflect the organization’s core values.” Items were rated on a 5-point Likert scale ranging from 1 (strongly disagree) to 5 (strongly agree). The Cronbach’s alpha for this scale was 0.92. The composite reliability (CR) was 0.93 and the average variance extracted (AVE) was 0.54, exceeding recommended thresholds.

#### Organizational identification

It measured organizational identification using a 6-item scale developed by Mael and Ashforth (1992). Sample items include “When someone criticizes my organization, it feels like a personal insult” and “I am very interested in what others think about my organization.” Items were rated on a 5-point Likert scale ranging from 1 (strongly disagree) to 5 (strongly agree). The Cronbach’s alpha for this scale was 0.88. The composite reliability (CR) was 0.89 and the average variance extracted (AVE) was 0.51, exceeding recommended thresholds.

#### Person-organization matching

It measured person-organization matching using a 4-item scale developed by Cable and DeRue (2002). Sample items include “My personal values match my organization’s values” and “The things that I value in life are very similar to the things that my organization values.” Items were rated on a 5-point Likert scale ranging from 1 (strongly disagree) to 5 (strongly agree). The Cronbach’s alpha for this scale was 0.85. The composite reliability (CR) was 0.86 and the average variance extracted (AVE) was 0.55, exceeding recommended thresholds.

#### Employee innovative behavior

It measured employee innovative behavior using a 9-item scale developed by Scott and Bruce (1994). Sample items include “I search out new technologies, processes, techniques, and/or product ideas” and “I generate creative ideas.” Items were rated on a 5-point Likert scale ranging from 1 (never) to 5 (always). The Cronbach’s alpha for this scale was 0.91. The composite reliability (CR) was 0.92 and the average variance extracted (AVE) was 0.52, exceeding the recommended thresholds of 0.70 and 0.50, respectively. This scale captures idea generation, promotion, and realization as a unified second-order construct, consistent with Scott and Bruce's (1994) original conceptualization.

#### Control variables

This study controlled for several demographic variables that may influence employee innovative behavior, including gender, age, education level, tenure and position level. The preliminary study of this research has shown that these demographic factors can affect employees’ opportunities and motivations to engage in innovative behavior.

#### Analytical strategy

This study conducted hierarchical regression analyses using SPSS 26.0 to test our hypotheses. First, it examined the direct effects of value-based leadership on employee innovative behavior (H1) and organizational identification (H2). Second, it tested the mediating effect of organizational identification using the PROCESS macro (Model 4) with 5,000 bootstrap samples (Hayes, 2018). Third, it tested the moderating effect of person-organization matching using PROCESS Model 1. Finally, it tested the moderated mediation model using PROCESS Model 7 to examine whether person-organization matching moderates the indirect effect of value-based leadership on innovative behavior through organizational identification (H5).

To address potential common method bias, it employed several procedural remedies, including temporal separation of measurements, assurance of anonymity, and randomization of item order (Podsakoff et al., 2003). Additionally, it conducted Harman single-factor test, which showed that the first factor explained 28.4% of the variance, below the 50% threshold. Furthermore, this study employed a marker variable technique using a theoretically unrelated construct and a latent variable method to control for common method variance. Both analyses confirmed that common method bias does not seriously threaten our findings.

## Results

### Descriptive statistics and correlations

Table 2 presents the means, standard deviations, and correlations among the study variables. Value-based leadership was positively correlated with organizational identification (*r* = 0.45, *p* < 0.01) and employee innovative behavior (*r* = 0.38, *p* < 0.01). Organizational identification was also positively correlated with employee innovative behavior (*r* = 0.52, *p* < 0.01). These correlations provide preliminary support for our hypothesized relationships. Furthermore, discriminant validity was confirmed: the square root of AVE for each construct exceeded its correlations with other constructs (Fornell-Larcker criterion), and all HTMT ratios were below 0.85, indicating that the four constructs are empirically distinct.

**Table 2:** Means, standard deviations, and correlations among study variables.

Variable	M	SD	1	2	3	4	5	6	7	8
1. Gender	0.55	0.50	-							
2. Age	36.80	8.20	0.06	-						
3. Education	2.76	0.68	−0.04	0.12*	-					
4. Tenure	6.40	4.80	0.03	0.45**	0.16**	-				
5. Position level	1.86	0.72	0.05	0.28**	0.22**	0.35**	-			
6. Value-based leadership	5.18	0.95	0.04	0.10	0.14*	0.16**	0.18**	-		
7. Person-org matching	4.92	1.08	−0.03	0.08	0.09	0.11*	0.12*	0.42**	-	
8. Organizational identification	4.76	1.12	0.02	0.12*	0.10	0.14*	0.15*	0.45**	0.48**	-
9. Innovative behavior	4.68	1.05	0.04	0.09	0.08	0.12*	0.14*	0.38**	0.35**	0.52**

*N* = 412. Gender: 0, female; 1, male. *p < 0.05; **p < 0.01.

### Hypothesis testing

Table 3 presents the results of hierarchical regression analyses testing our hypotheses. As shown in Model 1, value-based leadership significantly and positively predicted employee innovative behavior (*β* = 0.38, *p* < 0.001), supporting H1. Model 2 shows that value-based leadership significantly and positively predicted organizational identification (*β* = 0.45, *p* < 0.001), supporting H2.

**Table 3:** Results of hierarchical regression analysis.

Variable	Model 1 (IB)	Model 2 (OI)	Model 3 (IB)	Model 4 (OI)	Model 5 (IB)
Intercept	2.32**	2.45**	1.68**	2.38**	1.65**
	(−0.26)	(−0.30)	(−0.26)	(−0.29)	(−0.25)
Control variables					
Gender	0.06	0.04	0.05	0.03	0.05
	(−0.08)	(−0.09)	(−0.07)	(−0.08)	(−0.07)
Age	0.01	0.02*	0.01	0.02*	0.01
	(−0.01)	(−0.01)	(−0.01)	(−0.01)	(−0.01)
Education	0.05	0.07	0.03	0.06	0.03
	(−0.05)	(−0.06)	(−0.05)	(−0.05)	(−0.05)
Tenure	0.03*	0.04*	0.02	0.04*	0.02
	(−0.01)	(−0.02)	(−0.01)	(−0.02)	(−0.01)
Position level	0.08*	0.09*	0.05	0.08*	0.05
	(−0.04)	(−0.04)	(−0.03)	(−0.04)	(−0.03)
Main effects					
Value-based leadership	0.38***	0.45***	0.22**	0.42***	0.21**
	(−0.06)	(−0.07)	(−0.06)	(−0.07)	(−0.06)
Person-org matching				0.32***	0.15**
				(−0.06)	(−0.05)
VBL × P-O matching				0.18**	0.09*
				(−0.05)	(−0.04)
Mediator					
Organizational identification			0.35***		0.34***
			(−0.05)		(0.05)
Model statistics					
*R* ^2^	0.22	0.28	0.35	0.35	0.38
Δ*R*^2^	-	-	0.13	-	0.16
*F*	18.56***	24.28***	32.45***	29.86***	36.28***

*N* = 412; IB, innovative behavior; OI, organizational identification; VBL, value-based leadership; P-O, person-organization; *p < 0.05; **p < 0.01; ***p < 0.001.

### Mediation analysis

To test H3 regarding the mediating role of organizational identification, it conducted a bootstrap mediation analysis using PROCESS Model 4. The results showed that the indirect effect of value-based leadership on employee innovative behavior through organizational identification was significant (indirect effect = 0.18, 95% CI [0.12, 0.25]), supporting H3. The direct effect of value-based leadership on innovative behavior remained significant after including the mediator (direct effect = 0.22, *p* < 0.001), indicating partial mediation.

### Moderation analysis

**Figure 2:**
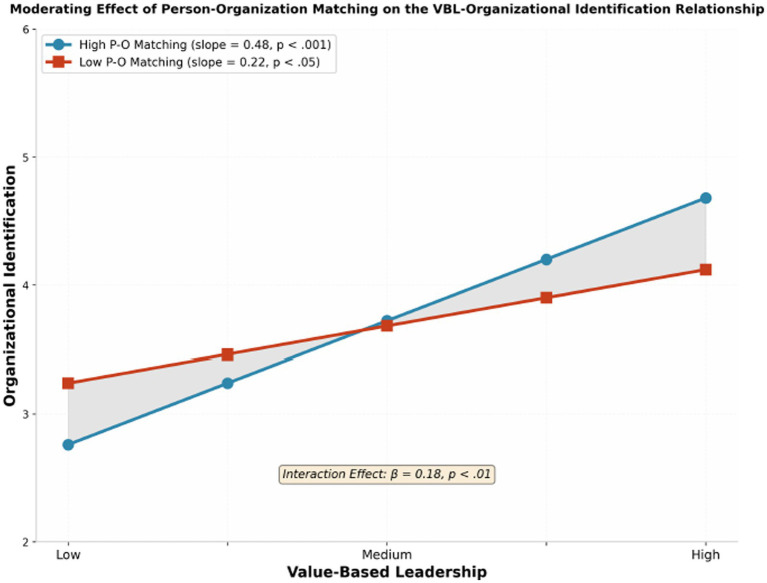
The moderating effect of person-organization matching.

To test H4 regarding the moderating effect of person-organization matching, it examined the interaction between value-based leadership and person-organization matching in predicting organizational identification. As shown in Model 2 of Table 3, the interaction term was significant (*β* = 0.18, *p* < 0.01), supporting H4. Figure 2 illustrates this interaction, showing that the positive relationship between value-based leadership and organizational identification is stronger when person-organization matching is high (+1 SD) compared to when it is low (−1 SD).

### Moderated mediation analysis

To test H5 regarding the moderated mediation effect, it examined whether person-organization matching moderates the indirect effect of value-based leadership on employee innovative behavior through organizational identification. Using PROCESS Model 5 with 5,000 bootstrap samples, it found that the index of moderated mediation was significant (index = 0.09, 95% CI [0.03, 0.14]), supporting H5.

Specifically, the indirect effect of value-based leadership on innovative behavior through organizational identification was stronger when person-organization matching was high (indirect effect = 0.24, 95% CI [0.16, 0.33]) compared to when it was low (indirect effect = 0.12, 95% CI [0.06, 0.19]). This pattern of results confirms that person-organization matching strengthens the mediating pathway from value-based leadership to innovative behavior via organizational identification.

Figure 1 presents the conceptual framework with the standardized path coefficients. The results provide comprehensive support for our hypothesized model, demonstrating the mediating role of organizational identification and the moderating role of person-organization matching in the relationship between value-based leadership and employee innovative behavior.

## Discussion

### Summary of findings

This study investigated the mechanisms through which value-based leadership is associated with employee innovative behavior, examining organizational identification as a mediator and person-organization matching as a moderator. Our findings provide strong support for the hypothesized model and offer several important insights into the leadership-innovation relationship. First, it found that value-based leadership positively predicts employee innovative behavior, consistent with previous research on the positive effects of value-based leadership on employee outcomes (Hendrickson and Blackmon, 2025). This finding suggests that leaders who articulate and embody organizational values can effectively inspire employees to engage in innovative behaviors that benefit the organization. The direct effect of value-based leadership on innovative behavior (*β* = 0.38) indicates that value-based leadership is a significant predictor of employee innovation, even after accounting for the mediating role of organizational identification.

Second, our results demonstrate that organizational identification partially mediates the relationship between value-based leadership and employee innovative behavior. This finding extends previous research by identifying a key psychological mechanism through which value-based leadership translates into innovative behavior. When leaders effectively communicate organizational values and create a shared sense of purpose, employees develop a stronger identification with the organization, which in turn motivates them to engage in innovative behaviors. The indirect effect through organizational identification (indirect effect = 0.18) suggests that approximately 43% of the total effect of value-based leadership on innovative behavior is transmitted through this mediating pathway. This mediating role of organizational identification is consistent with social exchange theory, which suggests that employees reciprocate favorable treatment from leaders through positive work attitudes and behaviors (Bhattacharjee and Sarkar, 2026).

Third, it found that person-organization matching moderates the relationship between value-based leadership and organizational identification. The positive effect of value-based leadership on organizational identification is stronger when employees perceive high levels of person-organization matching. This finding highlights the importance of value congruence in leadership effectiveness and suggests that value-based leadership may be particularly impactful in organizations where employees already share organizational values. The interaction effect (*β* = 0.15) indicates that the relationship between value-based leadership and organizational identification is significantly amplified when person-organization matching is high.

Finally, our results demonstrate that person-organization matching moderates the indirect effect of value-based leadership on employee innovative behavior through organizational identification. This moderated mediation finding provides a more nuanced understanding of when and how value-based leadership influences innovative behavior, highlighting the boundary conditions that shape the effectiveness of leadership interventions. The conditional indirect effects show that the mediating pathway through organizational identification is substantially stronger when person-organization matching is high (indirect effect = 0.24) compared to when it is low (indirect effect = 0.12).

### Theoretical implications

This study makes several important contributions to the leadership and innovation literature. First, by examining organizational identification as a mediator, this study extends understanding of the psychological mechanisms through which value-based leadership influences employee outcomes. While previous research has established positive relationships between value-based leadership and various employee attitudes and behaviors, the specific pathways through which these effects occur have received less attention. Our findings demonstrate that organizational identification plays a crucial mediating role, providing insights into the psychological processes underlying leadership effectiveness. This contribution is particularly important given the growing recognition that understanding mediating mechanisms is essential for advancing leadership theory and practice.

Second, by investigating person-organization matching as a moderator, it contributes to the boundary conditions literature on leadership effectiveness. Our findings suggest that the impact of value-based leadership is not uniform across all employees but depends on the degree of value congruence between individuals and organizations. This finding aligns with the person-environment fit perspective (Kristof-Brown et al., 2005) and highlights the importance of considering individual differences when examining leadership effects. The moderated mediation results provide a more comprehensive understanding of when value-based leadership is most effective in fostering organizational identification and innovative behavior.

Third, our study integrates social exchange theory and self-determination theory to explain how value-based leadership fosters employee innovative behavior. This theoretical integration provides a comprehensive framework for understanding leadership effects on innovation and suggests that both extrinsic (social exchange) and intrinsic (self-determination) mechanisms are at play. By drawing on multiple theoretical perspectives, this study offers a more nuanced understanding of the leadership-innovation relationship that can guide future research. Future research can build on this integrated framework to examine additional mediators and moderators that shape the relationship between leadership and innovation.

Fourth, our findings extend the conservation of resources theory (Hobfoll, 1989) by demonstrating how value-based leadership can serve as a resource that enables employees to engage in innovative behavior. Organizational identification, as a psychological resource, mediates the relationship between value-based leadership and innovative behavior, while person-organization matching acts as a contextual factor that enhances the value of this resource. This extension of conservation of resources theory provides a theoretical foundation for understanding how leadership can facilitate employee innovation by building and protecting psychological resources.

### Practical implications

Our findings have several practical implications for organizations seeking to enhance employee innovative behavior. First, organizations should invest in developing value-based leadership capabilities among their managers. Leadership development programs should emphasize the importance of articulating clear values, demonstrating commitment to those values through actions, and creating a shared sense of purpose among employees. Training interventions can help leaders develop the skills necessary to communicate organizational values effectively, model value-consistent behavior, and inspire followers to embrace shared values. By developing value-based leaders, organizations can foster the psychological conditions that promote employee innovation.

Second, organizations should pay attention to the value alignment between employees and the organization during the recruitment and selection process. Hiring employees whose personal values align with organizational values can enhance the effectiveness of value-based leadership interventions. Organizations can assess value fit through structured interviews, value assessments, and realistic job previews that communicate organizational values clearly. Additionally, organizations should communicate their values clearly during onboarding and reinforce these values throughout employees’ tenure to strengthen organizational identification and support innovative behavior.

Third, leaders should be aware that the effectiveness of their value-based leadership may vary depending on employees’ perceptions of person-organization matching. For employees who perceive high value congruence, value-based leadership is likely to be particularly effective in fostering organizational identification and innovative behavior. For employees who perceive low value congruence, leaders may need to employ additional strategies to build identification and promote innovation. This may include engaging in more frequent communication about organizational values, providing opportunities for employees to connect their personal values to organizational goals, and addressing value conflicts that may arise.

Fourth, organizations should consider implementing human resource practices that support value-based leadership and foster organizational identification. Performance management systems can be designed to recognize and reward innovative behavior, while also reinforcing organizational values. Compensation and recognition programs can be aligned with value-based behaviors to reinforce the importance of values in organizational life. Additionally, organizational culture initiatives can be implemented to strengthen shared values and create a climate that supports innovation and identification.

### Limitations and future research directions

This study has several limitations that should be acknowledged. First, convenience sampling approach may introduce selection bias, as participants were recruited through professional networks and online platforms, potentially limiting representativeness. Although it employed a two-wave design to establish temporal precedence, our data are correlational and do not allow for definitive causal inferences. This study has therefore used correlational language throughout the manuscript and caution readers against interpreting our findings as causal. The relationships observed in this study could potentially be explained by reverse causality or third variables not included in our model. The relationships observed in this study could potentially be explained by reverse causality or third variables not included in our model. Future research could employ experimental designs, such as leadership interventions or field experiments, to establish causality more definitively. Additionally, longitudinal designs with multiple measurement points could help clarify the temporal dynamics of the relationships examined in this study.

Second, our sample was drawn from organizations in China, which may limit the generalizability of our findings to other cultural contexts. Cultural values, such as collectivism, power distance, and uncertainty avoidance, may influence how employees respond to value-based leadership and how person-organization matching affects organizational identification. Future research should replicate our findings in different cultural settings to establish cross-cultural validity and examine whether cultural values moderate the relationships observed in this study.

Third, this study focused on organizational identification as a single mediator. While our theoretical framework and empirical results support the mediating role of organizational identification, other mechanisms may also be at play. Future research could examine additional mediators, such as psychological empowerment, creative self-efficacy, intrinsic motivation, or leader-member exchange quality, to provide a more comprehensive understanding of how value-based leadership influences innovative behavior. Multiple mediation analyses could help disentangle the relative contributions of different mediating mechanisms.

## Conclusion

This study provides valuable insights into the mechanisms through which value-based leadership is associated with employee innovative behavior. By examining organizational identification as a mediator and person-organization matching as a moderator, this study suggests that value-based leadership is positively related to innovative behavior both directly and indirectly through enhanced organizational identification. Furthermore, it shows that these relationships are strengthened when employees perceive high levels of person-organization matching. These findings contribute to the leadership and innovation literature by illuminating the psychological mechanisms and boundary conditions of value-based leadership’s influence on employee innovation. Organizations seeking to foster innovative behavior should invest in developing value-based leadership capabilities and attend to the value alignment between employees and the organization.

## Data Availability

The data analyzed in this study can be made available on request. Requests to access these datasets should be directed to the corresponding author.
